# Control values of intraocular pressure in different species: a review of literature

**DOI:** 10.3325/cmj.2024.65.518

**Published:** 2024-12

**Authors:** Maja Bakula, Tomislav Kuzman, Milan Radoš, Katarina Starčević, Ivana Jurjević, Marija Mamić, Boris Pirkić, Marijan Klarica

**Affiliations:** 1 Department of Ophthalmology, University of Zagreb School of Medicine, University Hospital Center Zagreb, Zagreb, Croatia; 2 Croatian Institute for Brain Research, University of Zagreb School of Medicine, Zagreb, Croatia; 3 Department of Neurology, University of Zagreb School of Medicine, University Hospital Center Zagreb, Zagreb, Croatia; 4 School of Veterinary Medicine, University of Zagreb, Zagreb, Croatia; 5 Department of Pharmacology and Croatian Institute for Brain Research, University of Zagreb School of Medicine, Zagreb, Croatia

## Abstract

It is generally accepted that intraocular pressure (IOP) depends on the rate of aqueous humor production, system outflow resistance, and episcleral venous pressure. Therefore, control IOP values are expected to be within the strict and predictable limits in specific animal species, and there should be no vast differences between species. However, in the literature the control IOP values significantly vary (from potentially “hypotensive” to “hypertensive”) within the same species, and especially between species depending on the measurement technique, head position in relation to the rest of the body, circadian rhythm, age, and topical and systemic drugs (anesthetics) applied. These variations make it difficult to compare different therapeutic approaches for intraocular hypertension, investigate the correlation between IOP and intracranial pressure, and determine target IOP values in glaucoma research. We recommend that different IOP physiology and pathophysiology studies take into account all the mentioned factors when describing IOP measurement methodology.

It is generally considered that aqueous humor pressure depends on the amount of its production and absorption (1). Thus, aqueous humor is believed to be actively produced by secretion through the epithelial cells of the ciliary body (which stems from the same neuroectodermal origin as the brain choroidal plexus), then flows from the posterior to the anterior chamber of the eye to be absorbed through the trabecular meshwork and Schlemm canal (conventional outflow) or through the uveal tissues (unconventional outflow) (2-4). Its formation has a hydrostatic and a secretory component. The hydrostatic component results from passive leakage of fluid from the blood, and the secretory component results from the active transport of sodium and other ions by the ciliary epithelium. Fluid volume in the aqueous system of the human eye is around 250 μL, with the formation rate of 2.5 μL/min and the turnover rate of 1.0%-1.5% per min (3).

IOP depends on the venous pressure, the rate of secretion, and resistance to fluid drainage, which can be expressed with the formula: IOP = ([F – Fu]/Ctot) + Pv (where F is aqueous humor formation rate, Fu is uveoscleral outflow rate, Ctot is total outflow facility, or total outflow conductance, and Pv is episcleral venous pressure) (5). This formula is known as the Goldmann equation, with various modifications.

The mean IOP in the human adult population is estimated at 15-16 mm Hg, with a standard deviation of nearly 3.0 mm Hg, ie, two standard deviations above the mean (6). These reference ranges date back to 1958, as reported in the study by Leydhecker et al (7). The Beijing Eye Study from 2011 (8) found normal IOP to be 14.5 ± 2.7 mm Hg with ±2 standard deviations (SD). This finding is consistent with the normal IOP range for the white population reported by other researchers. The researchers also listed systemic and ophthalmic factors that should be taken into account when determining IOP reference range: blood pressure, pulse rate, age, refractive error, central corneal thickness, and corneal curvature, among others (8-10). Conversely, there are still no exact reference intervals defined for commonly used experimental animals, despite numerous studies reporting control IOP values.

IOP can be measured invasively and non-invasively. An invasive method, used only in experimental animals, is the cannulation of the anterior chamber. The most frequently used non-invasive methods are Tono-Pen, TonoVet, Goldmann applanation tonometry, Schiotz indentation tonometry, and pneumotonometry. Newer tonometers, such as TonoLab and iCare TonoVet, are specially designed for IOP measurement in animals, while some older tonometers require modification of IOP using conversion tables (11).

A preliminary analysis of IOP control values in human and veterinary ophthalmology textbooks and review papers suggested that control values varied in individual species as a mean value ±2 SD. However, despite the use of appropriate techniques within the same animal species, in some papers there was a large dispersion of the results. Namely, the values ranged from potentially hypotensive to hypertensive. Therefore, in this study, we more systematically reviewed the current literature covering IOP measurement in control settings. Additionally, we investigated the contributing and confounding factors that should be addressed when comparing the results. We also highlighted some clinical entities (correlation between intracranial pressure and IOP, spaceflight-associated neuro-ocular syndrome, normotensive glaucoma) whose research requires defined reference points in order to compare the interrelationship of pressures.

## Methods

The current review involved the available literature published up to December 1, 2023, with no date restrictions. PubMed, Embase, and Web of Science were searched by using the MeSH keywords: “intraocular pressure” and selected animal species (˝cat,˝ ˝rabbit,˝ ˝monkey,˝ ˝mouse,˝ ˝rat,˝ ˝dog,˝ ˝donkey,˝ ˝horse˝) or “humans.” Inclusion criteria were IOP measurements in naive and untreated contralateral eyes, as in many studies the contralateral eye was considered an untreated internal control.

**Figure 1:**
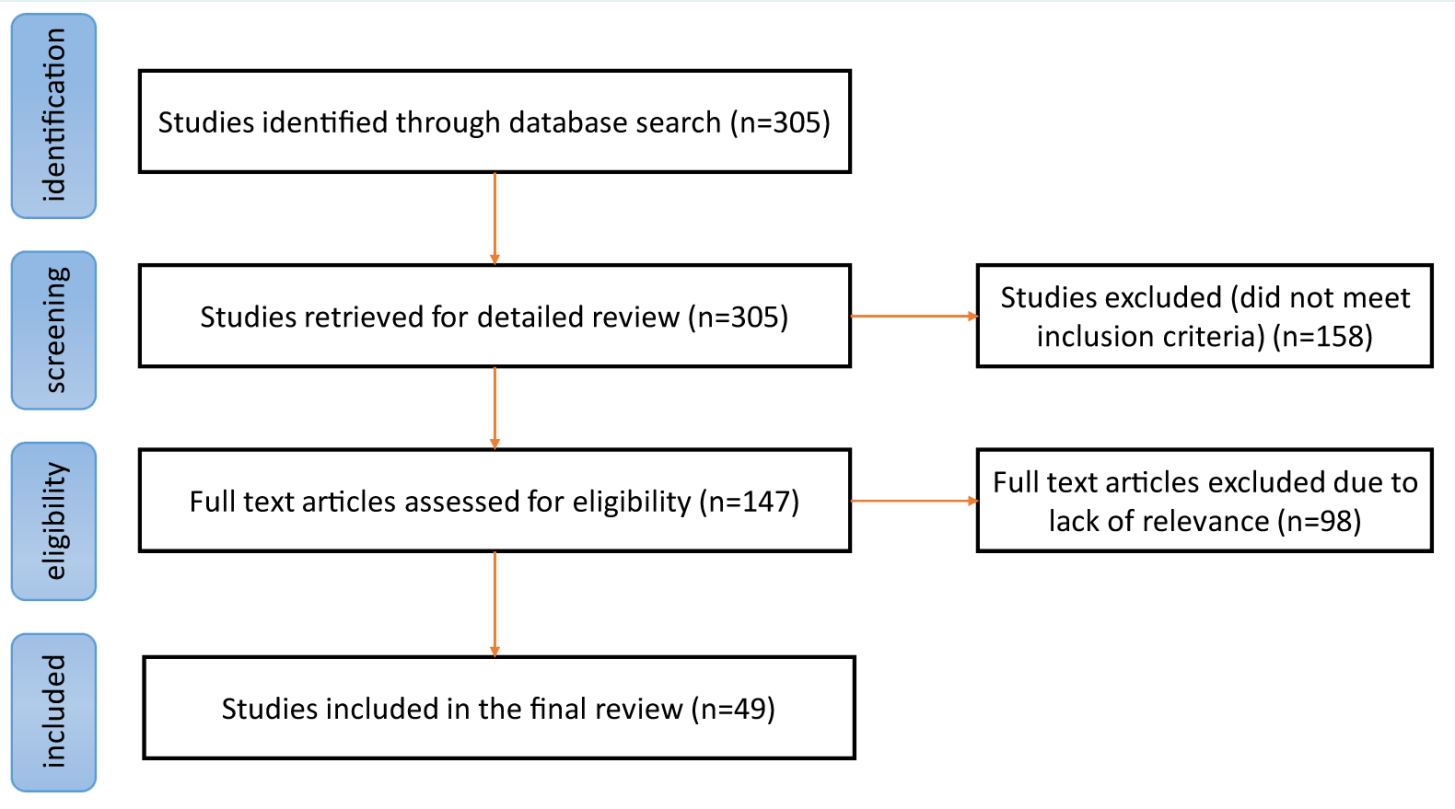
The PRISMA flowchart of the study selection process.

Publications were not included if they met any of the following criteria: treatment to the examined eye (vehicle or intravitreally treated eyes or sham controls) and genetically modified animals with genetically elevated intraocular pressure (ie, knockout animal strains, animals with congenital glaucoma) (Figure 1). We considered 305 articles, but due to a large number of publications, we included only those with an adequate number of examined eyes (“n”) for a specific species (for large experimental animals minimally n ≥4 eyes, for small animals minimally n ≥8 eyes). Studies in which data were shown graphically were excluded. Quantitative data were presented as mean ± standard deviation (SD), unless stated otherwise.

## Results

Table 1 presents control IOP values in nine animal species (cat, rabbit, monkey, mouse, rat, dog, miniature donkey, horse), in which IOP was measured with various techniques (cannulation of the anterior chamber, Goldmann applanation tonometry, Tono-Pen, pneumotonometry, and TonoVet). Reference IOP values reported for the same animal species highly varied depending on the measurement method.

**Table 1:** Control values of intraocular pressure (IOP) for different animal species (cat, rabbit, monkey, mouse, rat, dog, pig, miniature donkey, horse) obtained by different measurement methods (cannulation of anterior chamber, Goldmann applanation tonometry, Tono-Pen, pneumotonometry, TonoVet). The results are shown as mean value ± standard deviation of intraocular pressure in mmHg, unless stated otherwise; n = number of eyes

Animal species	Cat	Rabbit	Monkey	Mouse	Rat	Dog	Pig	Miniature donkey	Horse
Cannulation of anterior chamber	23.4 ± 2.8 (n = 5)^65¶^ 25.0 ± 3.0 (n = 10)^13*^	13.1 ± 1.3 (n = 6)^23†^ 14.1 ± 0.5 (n = 43)^70†^ 15.0 ± 6.3 (n = 8)^71^ 15.2 ± 0.8 (n = 14)^72†^ 18.5 ± 1.0 (n = 12)^72†^ 16.4 ± 1.1 (n = 25)^73*^ 18.1 ± 3.3 (n = 6)^24^	19.2 ± 0.9 (n = 11) ^79‡^	7.7 ± 0.5 (n = 8)^25†^ 9.4 ± 0.5 (n = 11)^25†^ 12.3 ± 0.5 (n = 10)^25†^ 13.7 ± 0.8 (n = 9)^25†^ 14.8 ± 2.2 (n = 173)^85^ 17.4 ± 0.6 (n = 60)^86†^ 17.8 ± 0.4 (n = 73)^87†^ 18.9 ± 2.0 (n = 8)^26^	10.6 ± 0.4 (n = 11)^89†^ 15.9 ± 0.4 (n = 20)^90†^		14.1 ± 2.2 (n = 7)^96^		
Goldmann		18.1 ± 1.7 (n = 4)^74*^	16.5 ± 2.8 (n = 5)^80^ 16.8 ± 0.5 (n = 14)^81†^	15.2 ± 0.6 (n = 22)^88†^					
Tono-Pen	12.3 ± 4.0 (n = 1068)^12^ 16.8 ± 3.6 (n = 50)^66^ 19.7 ± 5.6 (n = 37)^21^	5.8 ± 0.6 (n = 14)^15^ 9.7 ± 1.8 (n = 47)^75^ 11.5 ± 4.6 (n = 50)^76^ 11.8 ± 3.7 (n = 24)^107^	10.8 ± 1.7 (n = 41)^17^ 15.9 ± 2.8 (n = 20)^82^ 19.2 ± 4.2 (n = 24)^18^		9.9 ± 1.2 (n = 20)^22^ 12.8 ± 2.7 (n = 120)^91^ 20.8 ± 0.2 (n = 10)^92†^ 29 ± 2 (n = 16)^20^	12.8 ± 2.9 (n = 50)^94^ 14.0 ± 3.1 (n = 116)^95^	15.2 ± 1.8 (n = 5)^97^	20.7 ± 5.1 (n = 114)^99^	19.2 ± 4.7 (n = 72)^100^
Pneumo- tonometer	12.7 ± 1.1 (n = 12)^67†^ 17.9 ± 0.0 (n = 24)^68†^ 22.2 ± 1.2 (n = 12)^67†^	15.1 ± 2.3 (n = 15)^77^ 18.7 ± 0.4 (n = 31)^78†^ 20.5 ± 0.2 (n = 60)^16†^	14.9 ± 2.1 (n = 102)^83§^ 18.4 ± 0.6 (n = 12)^84†^		8.6 ± 1.3 (n = 61)^19^ 11.5 ± 0.7 (n = 105)^93^		12.6 ± 2.1 (n = 6)^98^		
TonoVet	20.6 ± 2.5 (n = 40)^68^ 18.9 ± 3.9 (n = 40)^106^	17.7 ± 3.1 (n = 50)^76^ 11.4 ± 3.9 (n = 24)^107^				15.0 ± 3.2 (n = 50)^94^ 15.3 ± 2.7 (n = 40)^106^		25.8 ± 5.7 (n = 114)^99^	25.7 ± 5.8 (n = 72)^100^ 22.2 ± 3.7 (n = 40)^106^

*mean ± standard error (SE).

†mean ± standard error of the mean (SEM).

‡IOP value expressed in mmH_2_O.

§mean IOP of the two eyes of each animal, one mean IOP value per animal

¶the superscript refers to the reference number.

Control IOP in cats varied from 12.3 ± 4.0 mm Hg measured with Tono-Pen (12) to 25.0 ± 3.01 mm Hg measured with the cannulation of the anterior chamber (13), which represents a range from potentially hypotensive to potentially hypertensive values (14). Very wide ranges were also observed in other animal species. In the rabbit, the values ranged from 5.8 ± 0.6 with Tono-Pen (15) to 20.5 ± 0.2 mm Hg with a pneumotonometer (16). In the monkey, they ranged from 10.8 ± 1.7 mm Hg (17) with TonoPen (18) to 19.2 ± 4.2 mm Hg with the cannulation of the anterior chamber. In the rat, the range was from 8.6 ± 1.3 mm Hg with a pneumotonometer (19) to 29 ± 2 mm Hg with TonoPen (20).

Furthermore, control values varied significantly even when they were measured with the same method within the same species (Table 1). For example, control values of IOP in cats obtained with Tono-Pen differed from 12.3 ± 4.0 mm Hg (12) to 19.7 ± 5.6 mm Hg (21). In rats, the interval was even wider, ranging from 9.9 ± 1.2 mm Hg (22) to 29.0 ± 2.0 mm Hg (20). When measured with anterior chamber cannulation, considered one of the most precise methods, in rabbits the values spanned from 13.1 ± 1.3 mm Hg (23) to 18.1 ± 3.3 mm Hg (24), while in mice the range was even wider – from 7.7 ± 0.5 (25) to 18.9 ± 2.0 mm Hg (26).

In healthy humans, control IOP values varied less when non-invasive techniques such as the Goldmann applanation tonometer and Tono-Pen were used in a sitting position (Table 2). This observation can be attributed to technical improvements of newer portable tonometers or a lack of confounding factors often found in research on laboratory animals, which will be further discussed in the text.

**Table 2:** Reference values of intraocular pressure in humans obtained with Goldmann applanation tonometer and Tono-Pen. Results are shown as mean value ± standard deviation of intraocular pressure in mmHg

Goldmann applanation tonometer	Tono-Pen	Number of eyes	Study
12.22 ± 3.19	16.87 ± 4.42	259	Okudo et al (101)
16.10 ± 3.07	16.71 ± 3.09	255	Dervisogullari et al (102)
14.0 ± 2.7	17.3 ± 3.8	92	Osman et al (103)
15.8 ± 4.3	17.7 ± 1.5	274	Magela-Vieira et al (104)
15.5 ± 2.2	16.1 ± 3.0	200	Yilmaz et al (105)

## Discussion

This review showed that IOP in experimental animals depended on the measurement technique (Table 1). Control results widely varied with different methods but also when the same method was used (Table 1). The reported reference values also varied between and within species, which would not be expected according to anatomical characteristics and physiology within the same species.

This clearly suggests that IOP control values are influenced by various factors, such as applied anesthetics, circadian rhythm, body and head position, fixation, age, animal species and strains, as well as the measurement method. In humans, additional factors have been identified: exercise, respiration, fluid intake, heart rate, blood flow, and topical and systemic medications (8,10). Even though the factors that influence IOP values are discussed in the literature (27,28), all of these factors are usually not reported in detail when the methodology and conditions for IOP measurement are described.

These factors could lead to significant variations in the measured values, and their influence could plausibly explain the observed high variability of control IOP values within the same species measured with the same method (Table 1).

### Anesthetics

In mice, anesthesia had an IOP-lowering effect that varied between strains, and also had a stronger effect on mice with higher baseline IOP (29). Some types of general anesthesia increased IOP in mice; however, it stabilized 10-15 minutes after induction (30). IOP in laboratory mice was 2 mm Hg lower with intraperitoneal than with gas anesthesia (31). Similarly, in laboratory rabbits intravenous and intramuscular anesthetics decreased IOP (32). In children, a recent systematic review showed that most anesthetic agents significantly decreased IOP over time after the induction phase of general anesthesia (33).

### Circadian rhythm

In rats, the baseline awake light and dark IOPs were 20.2 ± 2.1 and 30.4 ± 2.7 mm Hg, respectively (34). IOP varies to some extent due to pulsatility and circadian rhythm. The production of aqueous humor, for example, is higher during daytime and lower at night, possibly due to the diurnal activity of the sympathetic system (35,36). Aqueous humor flow rate and outflow facility are both reduced at night (36). Outflow facility is reduced at night, so that the mean IOP is usually 3 to 5 mm Hg higher at night than during the day (37). These results suggest that drainage reduction is more significant for IOP regulation than the rate of aqueous humor production. However, nocturnal changes in episcleral venous pressure and uveoscleral outflow probably contribute to lowering of IOP at night and maintaining its stability (38,39).

Newer research suggests that IOP follows a circadian rhythm synchronized with the suprachiasmatic nucleus, thought to be the circadian pacemaker. The suprachiasmatic nucleus resets peripheral clocks through sympathetic nerves or adrenal glucocorticoids. This suggests that IOP's circadian rhythm is governed by circadian time signals, sympathetic noradrenaline, and glucocorticoids, rather than the local clock (40).

### Body and head position

It is theoretically possible that the results related to circadian rhythm are simultaneously related to the body position, especially in humans, who sleep at night (horizontal plane) and are active during the day (head-up position). When the head is in an upright position, IOP is lower, and when it is in a supine position, it tends to rise. In humans, IOP is higher by 3-4 mm Hg in a supine than in an upright position due to increased episcleral venous pressure, regardless of the time of day (3).

In a rabbit model, IOP increased by 2.3 ± 0.4 mm Hg from a supine position to head-down tilt (41). In cats, body position change from horizontal to upright decreased IOP from 18.5 ± 0.6 to 14.3 ± 0.1 cmH_2_O (28). In mice, head-down position increased both IOP and episcleral venous pressure (42).

**Figure 2:**
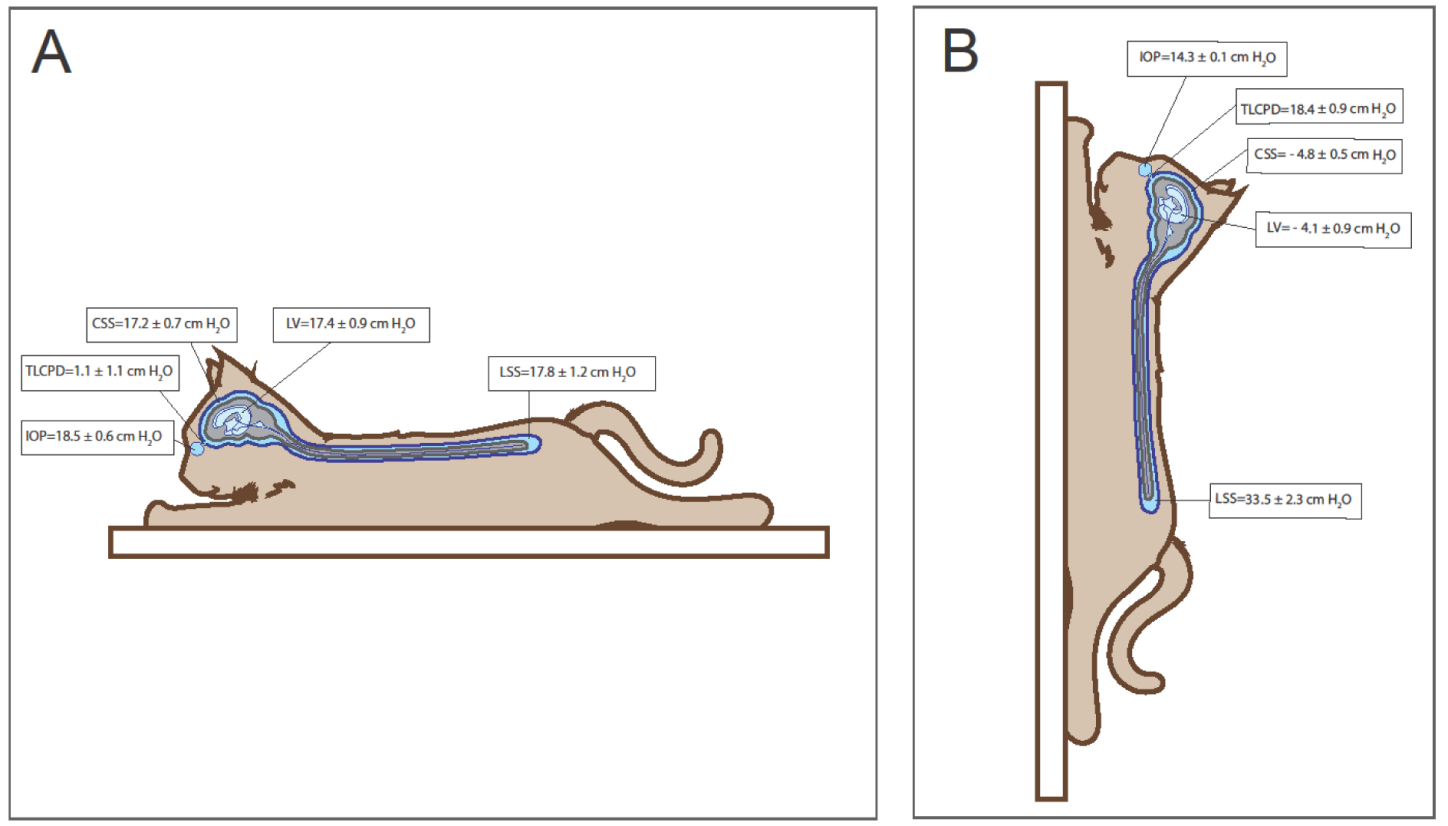
Intraocular pressure (IOP; cm H_2_O) and cerebrospinal fluid pressure (cm H_2_O) values in the cortical subarachnoid space (CSS), lateral ventricle (LV), and lumbar subarachnoid space (LSS) of the cat in the horizontal (**A**) and head-up position (**B**). Translamina cribrosa pressure difference (TLCPD) was also calculated. Pressure values are shown as mean ± standard error of the mean (data from 28).

Higher IOP values in a supine position can be explained with higher episcleral venous pressure and slower venous outflow from the head and the eye, a Valsalva-like phenomenon (37). They can also be explained with a postural oscillation of cerebrospinal fluid pressure (Figure 2). Higher cerebrospinal fluid pressure increases resistance to venous drainage from the eye (28,43).

### Measurement method

There are numerous studies that compare measurement techniques with the purpose of assessing the possibilities and usefulness of each. A suggestion is to set reference values of normal IOP for specific animal for each tonometer.

*Goldmann applanation tonometer*. Goldmann applanation tonometry (GAT) is considered to be a gold standard for IOP measurements in humans (44). However, the measurements are affected by the central corneal thickness, excessive or insufficient fluorescein in the tear film, high astigmatism, irregular or scarred cornea, squeezing of eyelids, wide pulse pressure, breath holding or other Valsalva maneuvers, pressure on the globe, excessive force applied to the restricted globe, vertical gaze, tight collars, repeated readings over a short period, and incorrect calibration.

In dogs, cats, and rabbits, the required diameter of the area of corneal applanation is 4 mm. In rats, the diameter is 2 mm, achieved with a tonometer tip with biprism angles of 48°, and the applied weight of 25 mg per Goldmann scale division (2 g full scale). In mice, the area of applanation is 1.5 mm in diameter, obtained with 36° biprism angles with the same applied weight as in rats (45-47).

*Perkins tonometer*. The Perkins tonometer can be used in either an upright or supine position and during anesthesia. Its disadvantages are low accuracy and the need for a more experienced examiner. In rats, the Perkins tonometer was calibrated against direct manometry, and normal values of IOP were detected (48), while in rabbits it underestimated IOP and had lower accuracy and higher variability (49).

*Tono-Pen*. Tono-Pen is a handheld electronic contact tonometer with the advantages of portability and reasonable accuracy in the eyes with distorted or edematous corneas. In monkeys, it provides reproducible measurements compared with the cannulation of the anterior chamber, and its measurement accuracy depends on the generation of an appropriate calibration curve (50). Tono-Pen was for a long time the preferred method in rats (51), but it is not much used anymore in rodents. It can be used to rapidly measure IOP in normal rabbit eyes, but it often underestimates pressures. However, a 95% confidence interval could be achieved with correction (52).

IOP values obtained by the Tono-Pen XL and Perkins tonometers in dogs and cats strongly correlated with those obtained by direct ocular manometry. No significant difference was found between the mean IOPs obtained with both tonometers in conscious animals, but the minimum and maximum values were on average 5-6 mm Hg higher with Tono-Pen XL. This justified the use of a table with normal values differentiated for each tonometer (53).

*Pneumotonometer*. A pneumotonometer´s accuracy is improved if an average of at least three readings is taken. Its advantage is measuring IOP on irregular corneal surfaces. In rabbits, it slightly overestimated IOP values (49).

*Rebound tonometers*. Rebound tonometry (eg, iCare, TonoVet, TonoLab) is commonly used in experiments on animals. In rabbits, TonoVet and Tono-Pen had excellent intrasession repeatability and inter-operator reproducibility but good intersession reproducibility. Both correlated well with manometry, but also underestimated the manometric IOP (54). Tonovet is well tolerated and thus a valuable alternative to conventional tonometers for clinical use in dogs and cats. Compared with the Tonopen Vet applanation tonometer, the use of TonoVet resulted in slightly elevated IOP readings if the tonometer was directed onto the peripheral cornea (approximately 1.5 mm from the limbus) and if the measuring distance was reduced to <4 mm. IOP was substantially underestimated with an angular deviation of the measuring axis (55).

Additionally, IOP values obtained with TonoPen need to be calibrated for use in rodents as its raw readings are only accurate in humans (56). These potential sources of error should be considered to avoid false IOP values.

TonoLab is now being widely used in IOP measurements in laboratory mice and rats. Millar et al provided a good review offering additional information on noninvasive measurement of IOP in laboratory animals with possible future directions (57).

IOP measurements with different tonometers in humans show conflicting results. This could be a consequence of additional uncontrolled factors, such as ocular hypertension, age, central corneal thickness, and IOP level (58). A recent meta-analysis including 22 primary studies from 15 countries published from 2011-2021 showed that IOP values in healthy adult population were marginally higher when measured with Tono-Pen compared with GAT, but no significant difference was observed (59).

*Invasive methods*. Anterior chamber cannulation is one of the most precise methods for IOP measurement. A cannula is inserted into the anterior chamber and connected to a pressure transducer. Many of experimental measurements in mice, rats, rabbits, cats, dogs, pigs, and monkeys were obtained with this method. Although considered the most accurate, the method has high intra- and inter-animal variability. Furthermore, measuring IOP invasively is not practical, and comparison with non-invasive techniques is required. A reliable and reproducible method for measuring mouse IOP is the servo-null micropipette system (60).

### Implications for research and clinical work

In our research, we encountered the problem of comparing IOP with intracranial pressure and other physiological parameters (28,43). To resolve methodological issues arising from this problem, these measurements need to be done in the same hydrostatic and biophysical position.

Determining reference values can be of interest in research of spaceflight-associated neuro-ocular syndrome (61-63), intracranial hypertension or hypotension, and normal tension glaucoma with trans–lamina cribrosa pressure difference evaluation (64). Additionally, this raises the question regarding the referent IOP that should represent a target value for therapeutic measures.

### Conclusion

IOP reference values in experimental animals are important for scientific research but remain incompletely documented and show wide variability. Since many factors may influence the reference values of IOP, in order to efficiently compare studies, researchers should thoroughly describe conditions under which IOP was measured (body and head position, fixation of the animal and the head with possible impact of fixator on orbital pressure, age, animal species and strains, circadian rhythm, respiration, fluid intake, heart rate, blood flow, anesthetics, topical and systemic medications, and method for IOP recording). This is important not only for better comparison of published work on IOP and glaucoma, but also in multidisciplinary research such as ocular and cerebrospinal fluid physiology and pathophysiology.
